# Addressing meniscal deficiency part 2: An umbrella review of systematic reviews and meta‐analyses on meniscal scaffold‐based approaches

**DOI:** 10.1002/jeo2.12108

**Published:** 2024-07-24

**Authors:** Kevin A. Wu, Aaron D. Therien, Lulla V. Kiwinda, Christian J. Castillo, Stephanie Hendren, Jason S. Long, Annunziato Amendola, Brian C. Lau

**Affiliations:** ^1^ Department of Orthopaedic Surgery Duke University Durham North Carolina USA; ^2^ School of Osteopathic Medicine Campbell University Lillington North Carolina USA; ^3^ Duke University School of Medicine, Medical Center Library & Archives Durham North Carolina USA

**Keywords:** meniscal allograft transplantation, meniscal scaffold, osteoarthritis, scaffold‐based strategies, systematic review

## Abstract

**Purpose:**

Meniscal injuries are common in knee surgery and often require preservation techniques to prevent secondary osteoarthritis. Despite advancements in repair techniques, some patients undergo partial meniscectomy, which can lead to postmeniscectomy syndrome. To address these challenges, meniscal substitution techniques like scaffolds have been developed. However, a comprehensive synthesis of the existing evidence through an umbrella review is lacking.

**Methods:**

A comprehensive search was conducted in the MEDLINE, Embase and Scopus databases to identify relevant systematic reviews and meta‐analyses. Studies were screened based on predefined inclusion and exclusion criteria. The quality of included studies was assessed using the AMSTAR‐2 tool.

**Results:**

A total of 17 studies met the inclusion criteria and were included in the review. Most studies focused on the use of collagen‐based scaffolds, with fewer studies evaluating synthetic scaffolds. The majority of studies (52.9%) were rated as having ‘Critically Low’ overall confidence, with only one study (5.9%) rated as ‘High’ confidence and most studies exhibiting methodological limitations, such as small sample sizes and lack of long‐term follow‐up. Despite these limitations, the majority of studies reported positive short‐term outcomes, including pain relief and functional improvement, following scaffold implantation. However, some studies noted a relatively high failure rate. Radiographically, outcomes also varied, with some studies reporting morphological deterioration of the implant seen on MRI, while others noted possible chondroprotective effects.

**Conclusions:**

Meniscal scaffold‐based approaches show promise in the management of meniscal deficiency; however, the current evidence is limited by methodological shortcomings. One notable gap in the literature is the lack of clear guidelines for patient selection and surgical technique. Future research should focus on conducting well‐designed randomized controlled trials with long‐term follow‐up to further elucidate the benefits and indications of these techniques in clinical practice. Additionally, efforts should be made to develop consensus guidelines to standardize the use of meniscal scaffolds and improve patient outcomes. Despite limited availability, synthesizing the literature on meniscal scaffold‐based approaches is crucial for understanding research, guiding clinical decisions and informing future directions.

**Level of Evidence:**

Level IV.

**Abbreviations**
ACLR: anterior cruciate ligament reconstruction
AMSTAR‐2: A MeaSurement Tool to Assess systematic Reviews 2
CMI: collagen meniscal implant
FDA: Food and Drug Administration
HTO: high tibial osteotomy
IKDC: International Knee Documentation Committee
KOOS: Knee injury and Osteoarthritis Outcome Score
MAT: meniscal allograft transplantation
MRI: magnetic resonance imaging
PRISMA: preferred reporting items for systematic reviews and meta‐analyses
PROMs: patient‐reported outcome measures
RoB: risk of bias
VAS: Visual Analog Scale

## INTRODUCTION

Meniscal injuries are common indications for knee surgery, often requiring preservation techniques to prevent secondary osteoarthritis [16]. Despite advancements in repair techniques, success often hinges on the tear's location and vascularity, leading some patients to undergo partial meniscectomy, which can contribute to postmeniscectomy syndrome due to reduced cartilage congruency [16, 18]. To address these challenges, meniscal substitution options like meniscal allograft transplantation (MAT) and scaffolds have been developed [13, 28]. MAT is typically reserved for total or subtotal meniscectomy cases, while scaffolds are preferred for partial defects requiring an intact meniscal rim and both anterior and posterior horns [7, 30].

These scaffolds, such as the Collagen Meniscal Implant (CMI), Actifit and NUsurface, act as templates for cellular ingrowth and tissue regeneration. CMI is sourced from bovine Achilles tendons to help facilitate cell ingrowth [22, 35]. Conversely, both Actifit and NUsurface, are synthetic implant alternatives with their polyethylene composition [1, 3, 14]. Despite these advancements, the evidence supporting these scaffold‐based approaches and their specific indications remains inconclusive [13]. There is a critical need to explore the unknowns in the landscape of meniscal scaffold‐based approaches, prompting a thorough examination of existing literature to pinpoint gaps in knowledge. Despite their limited availability in the market for medical use, synthesizing the literature on meniscal scaffold‐based approaches is crucial. It provides valuable insights into the current state of research, guides clinical decision‐making and informs future research directions.

This article constitutes part two of a two‐part umbrella review summarizing meniscal interventions [33]. Part two focuses on meniscal scaffold‐based approaches. The study's objectives are threefold: (1) to systematically review existing systematic reviews and meta‐analyses related to meniscal scaffold‐based approaches for deficiency; (2) to assess the quality, strengths and limitations of the published evidence in peer‐reviewed literature; and (3) to identify gaps in current research, thereby highlighting areas for future investigation. This umbrella review aims to conduct a systematic review of existing systematic reviews and meta‐analyses concerning meniscal scaffold‐based approaches, assess the quality of published evidence, and pinpoint areas of deficiency in current research.

## METHODS

The methodology for this umbrella Review of systematic reviews and meta‐analyses was detailed in a concomitant article (Part 1 of this Umbrella Review on Addressing Meniscal Deficiency) [33], adhering to the Preferred Reporting Items for Systematic Reviews and Meta‐Analyses (PRISMA) Statement guidelines for systematic reviews.

### Search strategy

The electronic literature search method used in this study has been detailed in the accompanying article (Part 1) [33]. It involved the same databases, search period, and keywords. The same independent reviewers (L. K., A. T.) manually screened the titles and abstracts of all the studies by using predetermined exclusion criteria. Any disagreement was resolved by the same third reviewer (K. W.).

### Inclusion and exclusion criteria

Before commencing the study, all authors agreed upon predetermined inclusion and exclusion criteria. Inclusion criteria encompassed studies that: (1) provided pertinent data on meniscal scaffold approaches, (2) were published in English‐language peer‐reviewed journals and (3) were systematic reviews and/or meta‐analyses. Exclusion criteria involved studies that: (1) lacked relevant outcomes meniscal scaffold approaches, (2) were abstract‐only studies and (3) were published in languages other than English.

The same template from the accompanying article (Part 1) [33] was used for data to extract citation details, study objectives and study outcomes for each included study.

### Quality of the studies

The methodological quality of the studies included in the analysis was evaluated using a summary table that outlined key aspects potentially affecting bias in the study of meniscal scaffold‐based approaches. This evaluation was conducted using the AMSTAR‐2 tool. Further details on this assessment can be found in Part 1 of this umbrella review [33]. Confidence in the results of each systematic review was determined by the AMSTAR‐2 tool and categorized as follows: ‘High’, ‘Moderate’, ‘Low’, or ‘Critically low’.

### Statistical analysis

Statistical analysis was conducted using R (version 3.1; The R Foundation) to calculate frequencies.

## RESULTS

**Figure 1:**
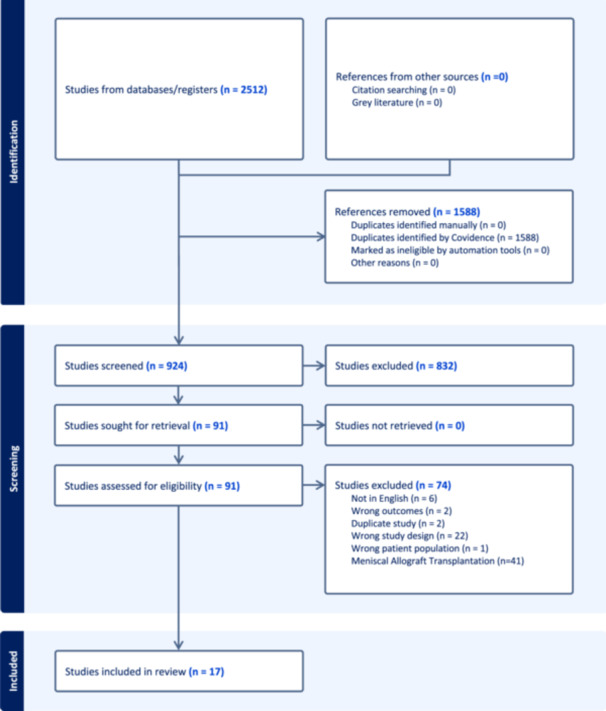
The preferred reporting items for systematic reviews and meta‐analyses (PRISMA) flowchart illustrates the screening process and selection of final articles for reviews focusing on meniscal scaffold‐based approaches.

A total of 2512 studies were initially identified through database searches and managed using EndNoteTM X9 (Clarivate Analytics). These references were then imported into Covidence for systematic review screening. After removing duplicates (1588 references), 924 studies underwent title and abstract screening, resulting in the exclusion of an additional 832 studies. The full text of 91 studies was thoroughly reviewed, leading to the exclusion of 32 articles for not meeting the predefined inclusion and exclusion criteria. Following the screening process, 58 studies were identified as focusing on meniscal allograft transplantation or meniscal scaffold‐based approaches. Among the 58 studies, 41 were excluded for focusing on meniscal allograft transplantation, resulting in 17 studies included in this umbrella review (Figure 1) [4, 7, 9, 10, 11, 13, 15, 19, 20, 21, 25, 26, 27, 29, 31, 32, 33, 34].

The primary reason for excluding full‐text articles (*n* = 41) was for focusing on meniscal allograft transplantation and were included in the accompanying article (Part 1) [33]. Most of the included studies were recently published, with 94.1% (16/17) published within the last decade (2013–2023), and 29.4% (5/17) published in the last two years (2021–2023). The oldest systematic review included in the analysis was published in 2012. Among the included studies, 3 (17.6%) were meta‐analyses and 14 (82.4%) were systematic reviews.

### Quality of the studies

The methodological quality of the 17 studies included in this review was assessed and summarized in Table 1. Using the AMSTAR‐2 criteria, the majority of studies (*n* = 9; 52.9%) were rated as having ‘Critically Low’ overall confidence [24]. Additionally, 5 reviews (29.4%) received a ‘Low’ confidence rating, while 2 reviews (11.8%) were rated as ‘Moderate’. Only 1 review (5.9%) was rated as ‘High’ confidence. These results indicate a widespread lack of confidence in the findings of most systematic reviews and meta‐analyses on meniscal scaffold‐based approaches, based on the AMSTAR‐2 quality rating criteria. Similar trends have been observed in other areas of orthopedic research and was demonstrated in our accompanying article on meniscal allograft transplantation (Part 1) [2, 23, 33].

**Table 1:** AMSTAR 2 assessment of the included studies.

	AMSTAR 2—Items																
References	1	2	3	4	5	6	7	8	9	10	11	12	13	14	15	16	Overall items
Bian et al. [4]	Yes	Yes	Yes	Yes	Yes	Yes	No	Yes	Yes	Yes	No MA	No MA	Yes	Yes	No MA	Yes	Low
Reale et al. [21]	Yes	Yes	Yes	Yes	Yes	Yes	Yes	Yes	Yes	Yes	Yes	Yes	Yes	Yes	Yes	Yes	High
Kohli et al. [13]	Yes	No	Yes	Yes	Yes	Yes	No	Yes	No	PY	No MA	No MA	Yes	Yes	No MA	No	Critically low
Veronesi et al. [31]	Yes	No	Yes	Yes	Yes	Yes	No	Yes	Yes	Yes	No MA	No MA	Yes	Yes	No MA	Yes	Critically low
Li et al. [15]	Yes	Yes	Yes	Yes	Yes	Yes	No	Yes	Yes	Yes	Yes	Yes	Yes	Yes	Yes	Yes	Low
Smoak et al. [26]	Yes	Yes	Yes	Yes	Yes	Yes	No	Yes	Yes	Yes	No MA	PY	Yes	Yes	No MA	Yes	Low
Ranmuthu et al. [20]	Yes	Yes	Yes	Yes	No	No	No	Yes	Yes	PY	No MA	No MA	Yes	Yes	No MA	No	Moderate
Shin et al. [25]	Yes	No	No	Yes	Yes	Yes	No	Yes	Yes	Yes	Yes	Yes	Yes	Yes	Yes	Yes	Low
Houck et al. [11]	Yes	Yes	Yes	Yes	Yes	No	No	Yes	No	Yes	No MA	No MA	No	No	No MA	Yes	Critically low
Vaquero and Forrial [29]	Yes	No	PY	No	No	No	No	Yes	No	No	No MA	No MA	No	Yes	No MA	Yes	Critically low
Tark et al. [27]	Yes	Yes	Yes	Yes	Yes	Yes	No	Yes	No	Yes	No MA	No MA	Yes	Yes	No MA	Yes	Critically low
Zaffagnini et al. [34]	Yes	No	Yes	Yes	Yes	Yes	No	Yes	No	No	No MA	No MA	No	Yes	No MA	No	Critically low
Warth and Rodkey [32]	Yes	No	Yes	Yes	Yes	Yes	No	Yes	No	Yes	No MA	No MA	No	Yes	No MA	Yes	Critically low
Filardo et al. [7]	Yes	Yes	Yes	Yes	Yes	Yes	No	Yes	No	No	No MA	No MA	No	No	No	No	Critically low
Grassi et al. [9]	Yes	Yes	Yes	Yes	No	No	Yes	Yes	No	No	No MA	No MA	Yes	Yes	Yes	No	Low
Papalia et al. [19]	Yes	No	Yes	Yes	Yes	Yes	No	Yes	No	Yes	No MA	No MA	No	Yes	No MA	Yes	Critically low
Harston et al. [10]	Yes	Yes	Yes	Yes	No	No	Yes	Yes	Yes	No	No MA	No MA	Yes	No	Yes	No	Moderate

*Note*: Description of AMSTAR‐2 Items: 1—Did the research questions and inclusion criteria for the review include the components of PICO?; 2—Did the report of the review contain an explicit statement that the review methods were established prior to the conduct of the review and did the report justify any significant deviations from the protocol?; 3—Did the review authors explain their selection of the study designs for inclusion in the review?; 4—Did the review authors use a comprehensive literature search strategy?; 5—Did the review authors perform study selection in duplicate?; 6—Did the review authors perform data extraction in duplicate?; 7—Did the review authors provide a list of excluded studies and justify the exclusions?; 8—Did the review authors describe the included studies in adequate detail?; 9—Did the review authors use a satisfactory technique for assessing the risk of bias (RoB) in individual studies that were included in the review?; 10—Did the review authors report on the sources of funding for the studies included in the review?; 11—If meta‐analysis was performed did the review authors use appropriate methods for statistical combination of results?; 12—If meta‐analysis was performed, did the review authors assess the potential impact of RoB in individual studies on the results of the meta‐analysis or other evidence synthesis?; 13—Did the review authors account for RoB in individual studies when interpreting/discussing the results of the review?; and 14—Did the review authors provide a satisfactory explanation for, and discussion of, any heterogeneity observed in the results of the review?; 15—If they performed quantitative synthesis did the review authors carry out an adequate investigation of publication bias (small study bias) and discuss its likely impact on the results of the review?; 16—Did the review authors report any potential sources of conflict of interest, including any funding they received for conducting the review?

Abbreviations: No MA, no meta‐analysis; PY, partial yes.

No articles were excluded from further analysis based on the quality assessment, as this study aims to identify areas for improvement in the quality of systematic reviews of meniscal scaffold approaches through a comprehensive review and assessment of the existing literature. The majority of studies 14 (82.4%) did not provide a list of excluded studies. There were approximately 52.9% of the studies did not adequately describe the technique used to assess the risk of bias (RoB) within the included studies. Furthermore, many studies failed to discuss or interpret the results in light of RoB (35.3%). There were six reviews (35.3%) that did not disclose any source of funding for the study.

### Studies characteristics

The 17 reviews included in the final review were published across 12 different journals. The top three journals where these studies were published were Knee Surgery, Sports Traumatology, Arthroscopy (KSSTA) (23.5%), Arthroscopy (11.8%) and International Orthopaedics (11.8%) (Table 2). Unlike the reviews surrounding meniscal allograft transplantation, there were no reviews published within the *American Journal of Sports Medicine (AJSM)*. The studies were conducted primarily in Italy (29.4%) and the United States (29.4%), followed by the United Kingdom (17.2%), and South Korea (11.8%). On average, these reviews included 15.7 studies, with a predominant focus on retrospective cohort studies (Table 3). The majority of reviews included studies that predominantly involved male participants, ranging from 60% to 80% [7, 9, 11, 13, 15, 19, 21, 32, 34].

**Table 2:** The most common journals and countries of origin.

	Studies (*n*)	Studies (%)
*Journal*		
Knee Surgery, Sports Traumatology, Arthroscopy	4	23.5
Arthroscopy: The Journal of Arthroscopic and Related Surgery	2	11.8
International Orthopaedics	2	11.8
*Country of Origin*		
United States	5	29.4
Italy	5	29.4
United Kingdom	3	17.6
South Korea	2	11.8

**Table 3:** Demographics of the included studies.

Study	Aim of study	Number of studies/participants	Age of participants, range/mean	Percentage male
Bian et al. [4]	To investigate the up‐to‐date clinical outcomes of tissue‐engineered meniscus implants for meniscus defects	19 studies, 974 patients	30–52 years	N/A
Reale et al. [21]	To compare the results of two meniscal scaffolds, CMI and Actifit, for the treatment of partial meniscal lesions	1276 patients	36.3 years	73.20%
Kohli et al. [13]	To compare clinical outcomes and failure rates of patients who have had implantation with Actifit, CMI, and NUsurface meniscal scaffolds	12 studies, 436 participants	36.7 years	70.18%
Veronesi et al. [31]	The aim of this systematic review was to collect and evaluate all the available evidence on biosynthetic scaffolds for meniscus regeneration both in vivo and in clinical studies	76 studies, 46 in vivo, 30 clinical	N/A	N/A
Li et al. [15]	Review the medium and long‐term follow‐up results after applying PU stent	613 patients	36 years	60%
Smoak et al. [26]	To provide a qualitative summary of the published systematic reviews and meta‐analyses regarding the meniscus	142 studies	Adults aged 40 and older	N/A
Ranmuthu et al. [20]	To assess whether the CMI and Actifit scaffolds, when used in clinical practice, improve clinical outcomes and demonstrate the ideal biological and biomechanical properties of scaffolds: Being chondroprotective, porous, resorbable, able to mature and promote regeneration of tissue	8 studies, 224 patients	N/A	N/A
Shin et al. [25]	This meta‐analysis was performed to assess clinical and MRI outcomes after surgery in patients with partial meniscal defects treated with PU meniscal scaffolds	18 studies, 489 patients	N/A	N/A
Houck et al. [11]	To evaluate the current literature in an effort to assess specific clinical outcomes following meniscal scaffold implantation using the two available scaffolds: Collagen Meniscal Implant (CMI) and the Actifit polyurethane meniscal scaffold	658 patients	36 years	72.74%
Vaquero and Forriol [29]	The aim of this study was to analyze the current state of meniscal surgery aimed at preserving morphology and conserving the biomechanics of the knee to prevent joint degeneration	N/A	N/A	N/A
Tark et al. [27]	The purpose of this study was to apply horizon scanning methods for predicting potential impacts of the implantation of polyurethane scaffold in Korean healthcare system and to assess the safety, effectiveness, and potential impacts of polyurethane scaffolds on patients or healthcare services in the near future	254 patients	N/A	N/A
Zaffagnini et al. [34]	The purpose was to evaluate, using the Genovese score, the MRI behavior of the CMI at different follow‐up periods and investigate possible differences in the behavior of lateral and medial CMI	6 studies, 194 patients	N/A	79%
Warth and Rodkey [32]	The purpose of this study was to evaluate the clinical and structural outcomes after resorbable collagen meniscus scaffold implantation	13 studies, 674 patients	Range: 31–44 years	80%
Filardo et al. [7]	To document the available clinical evidence to support meniscal scaffold implantation	613 patients	36.5 years	76.20%
Grassi et al. [9]	To summarise and evaluate the clinical outcomes of the collagen meniscus implant (CMI) and its complication and failure rates	342 patients	37.8 years	79%
Papalia et al. [19]	To understand if partial meniscus replacement really improves clinical outcome in patients undergoing a meniscectomy, following a meniscal injury and whether this procedure is more beneficial than partial meniscectomy alone, if it can be suggested as a safe procedure and if there are differences in the final results achieved with the use of the two classes of devices	15 studies, 624 patients	37 years	70.35%
Harston et al. [10]	To determine collagen meniscus implant (CMI) efficacy for improving patient function, symptoms, and activity level	N/A	N/A	N/A

### Scaffold‐based approaches

Twelve of the studies examined using a polyurethane‐based scaffold (Actifit; Orteq Bioengineering) (NUsurface Meniscus Implant) while 11 studies examined the use of the CMI (CMI; Ivy Sports Medicine). One implant, CMI, has been approved for use in the United States while Actifit has gotten breakthrough device designation by the United States Food and Drug Administration (FDA). At the time of this study, NUsurface has not been approved to be used United States but have been approved in Europe and Israel.

**Figure 2:**
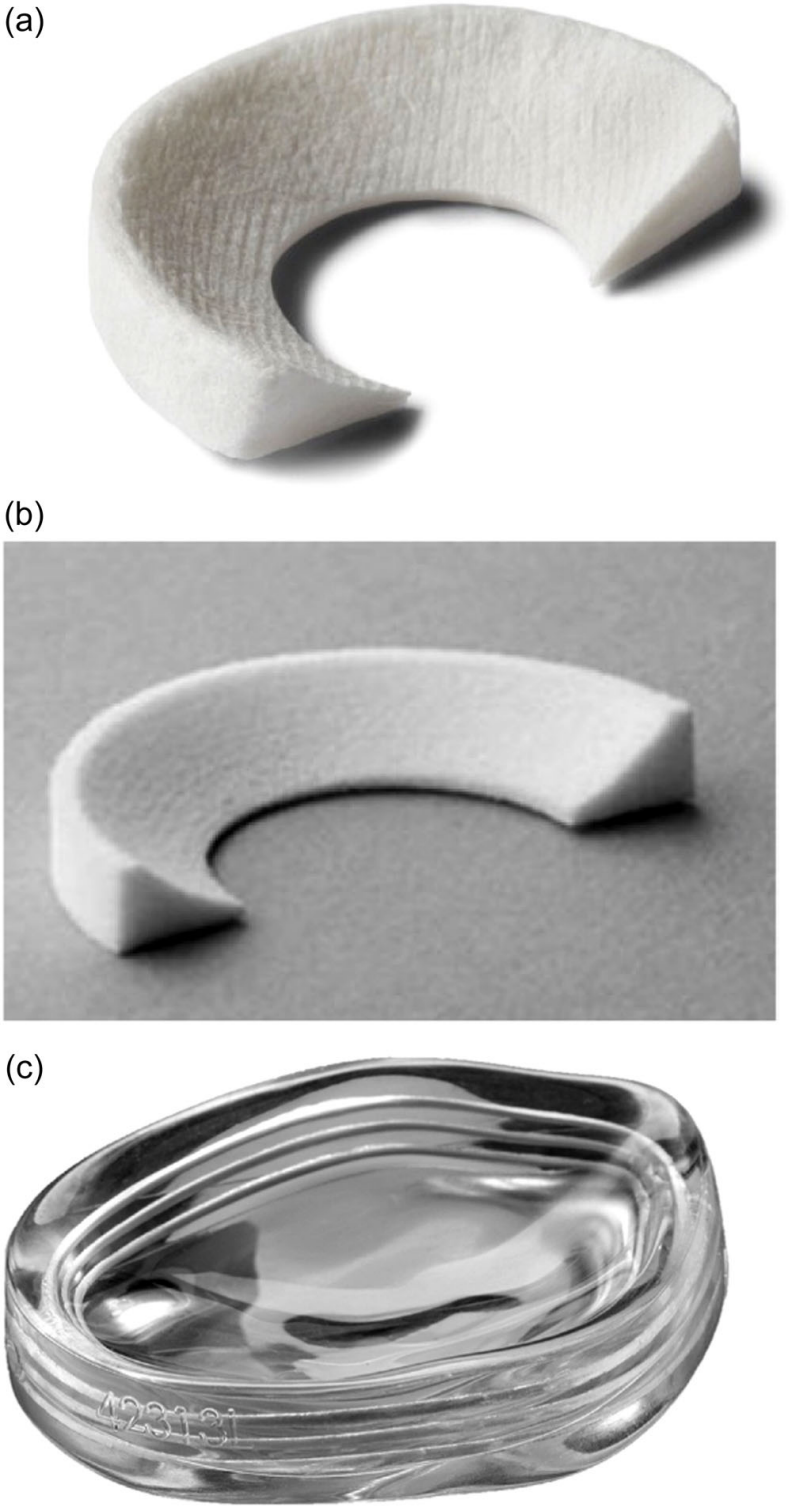
Various scaffold implants available: (a) collagen meniscus implant, (b) actifit implant (image adapted from Baynat and colleagues) and (c) NUsurface meniscus implant.

The three different implants are shown in Figure 2. The Actifit scaffold is a synthetic, biodegradable scaffold made from polyurethane, designed to promote meniscus regeneration. It features a mesh‐like structure to support this process. In contrast, the Collagen Meniscal Implant is made from highly purified collagen sourced from bovine Achilles tendon. It is intended to act as a scaffold for new tissue growth and is commonly used in meniscal repair and regeneration procedures. The NUsurface Meniscus Implant is a medical device designed to replace the damaged portion of the meniscus in patients with persistent knee pain following meniscus surgery. It is made from medical‐grade plastic and can be inserted into the knee joint without requiring additional surgery for attachment to the surrounding tissue.

Scaffold‐based repairs are employed to address meniscus defects following partial meniscectomy as they require a meniscal rim for attachment and the presence of both anterior and posterior horns [7, 21]. It's crucial to note that uncorrected malalignment is a contraindication for meniscal scaffold implantation, necessitating a combination of meniscal scaffold and osteotomy [8]. Scaffold approaches are recommended for treating symptomatic chronic lesions in properly aligned knees, supported by histologic and imaging evidence of tissue regeneration [7]. These scaffolds are contraindicated in advanced cases of osteoarthritis and the presence and quality of the cartilage should be appropriately assessed preoperatively [6]. Clear guidelines for the use of scaffolds have not been established [6].

### Clinical outcomes following scaffold implantation

Bian and colleagues examined the use of all three meniscal implants (Actifit, CMI, NUsurface) and concluded that the bioengineered implants can improve short‐term knee symptoms and function, but their long‐term benefits for meniscus defects remained uncertain [4] (Table 4). Reale and colleagues performed a metanalysis comparing 472 CMI versus 804 Actifit with generally low‐quality evidence and found that both scaffolds showed significant improvement in all clinical scores, with no differences between them in patient‐reported outcome measures, activity level, or risk of failure [21]. Kohli and colleagues study included 262 patients were treated with Actifit, 109 with CMI and 65 with NUsurface [13]. They concluded that based on the available evidence, using meniscal scaffolds as the only treatment for partial meniscal defects is not recommended due to the relatively high failure rate and lack of sufficient clinical data. They had reported that the Actifit scaffold had a failure rate of 18% (range 6.3%–31.8%) over an average follow‐up of 66.8 months, while the CMI had a failure rate of 6.5% (range 0%–11.8%) over an average follow‐up of 97.1 months. The NUsurface failure rate was 16.9% at 12 months. They noted that patients undergoing these procedures also had other concurrent procedures, which makes it challenging to isolate the effect of the scaffold alone. Similarly, Veronesi and colleagues, Harston and colleagues and Houck and colleagues examined CMI and Actifit and found that while short‐ to mid‐term outcomes had positive results, there were large limitations of the available clinical literature, including a high number of concurrent procedures and a lack of randomized trials [10, 11, 31]. Tark and colleagues looked at the early outcomes of polyurethane‐based scaffold in Korean studies and found that while there were promising short term results, the studies lacked any comparison control groups [27].

**Table 4:** Patient reported outcome measures, failure rate and reoperation rate for scaffold based interventions.

**Study**	**Intervention description**	**Base line PROMs**	**Postoperative PROMs**	**Mean follow‐up**	**Survivorship**	**Failure and reoperation rate**
Bian et al. [4]	CMI	VAS average: 4.3 VAS number of studies: 3 IKDC: N/A IKDC number of studies: 1 KOOS average: N/A KOOS number of studies: N/A Tegner average: 3.5 Tegner number of studies: 2 Lysholm average: 66 Lysholm number of studies: 3	VAS average: 2.1 VAS number of studies: 3 IKDC: N/A IKDC number of studies: 1 KOOS average: N/A KOOS number of studies: N/A Tegner average: 6 Tegner number of studies: 2 Lysholm average: 91 Lysholm number of studies: 3		Survivorship 1: Percentage: 96% Follow‐up: 2 years Survivorship 2: Percentage: 85% Follow‐up: 5 year	
Reale et al. [21]	Actifit	VAS average: N/A VAS number of studies: N/A IKDC: N/A IKDC number of studies: N/A KOOS average: N/A KOOS number of studies: N/A Tegner average: N/A Tegner number of studies: N/A Lysholm average: N/A Lysholm number of studies: N/A	VAS average: 2.22 VAS number of studies: 6 IKDC: 73.84 IKDC number of studies: 3 KOOS average: N/A KOOS number of studies: N/A Tegner average: 4.44 Tegner number of studies: 7 Lysholm average: 88.12 Lysholm number of studies: 5	(24–36) months		Failure rate: 0.09% (95% CI: 0.05, 0.16)
Kohli et al. [13]	Actifit	VAS average: 5.5 VAS number of studies: 3 IKDC: N/A IKDC number of studies: N/A KOOS average: N/AKOOS number of studies: 3 Tegner average: 3.6 Tegner number of studies: 2 Lysholm average: 50.6 Lysholm number of studies: N/A	VAS average: 2.1 VAS number of studies: 3 IKDC: N/A IKDC number of studies: N/A KOOS average: N/A KOOS number of studies: 3 Tegner average: 4.6 Tegner number of studies: 2 Lysholm average: 81.3 Lysholm number of studies: 2	66.8 months		Failure rate: 18% Number of studies: 5
Li et al. [15]	Polyurethane meniscal scaffold	VAS average: N/A VAS number of studies: N/A IKDC: N/A IKDC number of studies: N/A KOOS average: N/A KOOS number of studies: N/A Tegner average: N/A Tegner number of studies: N/A Lysholm average: N/A Lysholm number of studies: N/A	VAS average change: 2 point decrease VAS number of studies: 11 IKDC average change: 1.65 point increase IKDC number of studies: 12 KOOS average change: 24.95 point increase KOOS number of studies: 9 Tegner average change: 0.34 point increase Tegner number of studies: 7 Lysholm average change: 1.63 point increase Lysholm number of studies: 8	45 months		
Ranmuthu et al. [20]	CMI	VAS average: 5.5 VAS number of studies: 1 IKDC: N/A IKDC number of studies: N/A KOOS average: N/A KOOS number of studies: N/A Tegner average: 2.3 Tegner number of studies: 3 Lysholm average: 60.1 Lysholm number of studies: 3	VAS average: 1.9 VAS number of studies: 1 IKDC: N/A IKDC number of studies: N/A KOOS average: N/A KOOS number of studies: N/A Tegner average: 5 Tegner number of studies: 3 Lysholm average: 93.7 Lysholm number of studies: 3			
Shin et al. [25]	Polyurethane meniscal scaffold	VAS average: 4.34 VAS number of studies: 11 IKDC: 40.49 IKDC number of studies: 10 KOOS average: 52.24 KOOS number of studies: 9 Tegner average: 3.12 Tegner number of studies: 5 Lysholm average: 60.09 Lysholm number of studies: 7	VAS average: 2.14 VAS number of studies: 11 IKDC: 69.57 IKDC number of studies: 10 KOOS average: 79.54 KOOS number of studies: 9 Tegner average: 4.38 Tegner number of studies: 5 Lysholm average: 88.15 Lysholm number of studies: 7	30 months		
Houck et al. [11]	Actifit	VAS average: 5.6 VAS number of studies: 9 IKDC: 44 IKDC number of studies: 8 KOOS average: 61 KOOS number of studies: 7 Tegner average: 3 Tegner number of studies: 3 Lysholm average: 60 Lysholm number of studies: 4	VAS average: 2.1 VAS number of studies: 9 IKDC: 70 IKDC number of studies: 8 KOOS average: 83 KOOS number of studies: 7 Tegner average: 4 Tegner Number of studies: 3 Lysholm sverage: 82 Lysholm number of studies: 4	40 months		Failure rate: 9.9% Number of studies: 11
Tark et al. [27]	Polyurethane scaffold implant	VAS average: N/A VAS number of studies: 5 IKDC: N/A IKDC number of studies: 5 KOOS average: N/A KOOS number of studies: 5 Tegner average: N/A Tegner number of studies: 3 Lysholm average: N/A Lysholm number of studies: 4	VAS average: N/A VAS number of studies: 5 IKDC: N/A IKDC number of studies: 5 KOOS average: N/A KOOS number of studies: 5 Tegner average: N/A Tegner number of studies: 3 Lysholm average: N/A Lysholm number of studies: 4	Range from 3–24 months		
Warth and Rodkey [32]	CMI and polyurethane	VAS average: 4.6 VAS number of studies: 5 IKDC: N/A IKDC number of studies: N/A KOOS average: N/A KOOS number of studies: N/A Tegner average: 2.9 Tegner number of studies: 6 Lysholm average: 63.8 Lysholm number of studies: 7	VAS average: 1.8 VAS number of studies: 5 IKDC: N/A IKDC number of studies: N/A KOOS average: N/A KOOS number of studies: N/A Tegner average: 5.2 Tegner number of studies: 6 Lysholm average: 90.3 Lysholm number of studies: 7	3–152 months		
Filardo et al. [7]	CMI	VAS average: 3.1 VAS number of studies: 11 IKDC: 55.5 IKDC number of studies: 7 KOOS average: N/A KOOS number of studies: N/A Tegner average: 2.7 Tegner number of studies: 9 Lysholm average: 45.5 Lysholm number of studies: 13	VAS average: 1.5 VAS number of studies: 11 IKDC: 79.9 IKDC number of studies: 7 KOOS average: N/A KOOS number of studies: N/A Tegner average: 5.3 Tegner number of studies: 9 Lysholm average: 88.4 Lysholm number of studies: 13	48.2 months		Failure rate: 6% Number of studies: 14
Grassi et al. [9]	CMI	VAS average: 3.9 VAS number of studies: 8 IKDC: N/A IKDC number of studies: N/A KOOS average: N/A KOOS number of studies: N/A Tegner average: 3 Tegner number of studies: 7 Lysholm average: 63.3 Lysholm number of studies: 10	VAS average: 1.3 VAS number of studies: 4 IKDC: N/A IKDC number of studies: N/A KOOS average: N/A KOOS number of studies: N/A Tegner average: 6 Tegner number of studies: 4 Lysholm average: 86.3 Lysholm number of studies: 4			Failure rate: 3.8% Number of studies: 6 Reoperation rate: 6.8% Number of studies: 8
Papalia et al. [19]	Partial meniscal replacement	VAS average: N/A VAS number of studies: N/A IKDC: N/A IKDC number of studies: N/A KOOS average: N/A KOOS number of studies: N/A Tegner average: N/A Tegner number of studies: N/A Lysholm average: N/A Lysholm number of studies: N/A	VAS average: N/A VAS number of studies: 9 IKDC: N/A IKDC number of studies: 6 KOOS average: N/A KOOS number of studies: 3 Tegner average: N/A Tegner number of studies: 9 Lysholm average: N/A Lysholm number of studies: 12	56 months		

Abbreviations: CMI, collagen meniscus implant; IKDC, International Knee Documentation Committee; KOOS, Knee injury and Osteoarthritis Outcome Score; N/A, not available; VAS, Visual Analog Scale.

Eight reviews (57.1%) included studies where a concomitant procedure was performed alongside scaffold placement, while one review (7.1%) exclusively focused on studies involving scaffold placement alone. The most frequent concurrent procedures were ACLR, HTO and microfracture.

Twelve studies (70.6%) included patient‐reported outcome measures (PROMs), with the Lysholm score being the most commonly reported (*n* = 12; 70.6%), followed by the Tegner activity scale (*n* = 12; 70.6%), VAS for pain (*n* = 12; 70.6%), IKDC score (*n* = 8; 47.1%) and the KOOS (*n* = 6; 35.3%) [4, 7, 9, 11, 13, 15, 19, 20, 21, 25, 27, 32]. All reviews that reported on PROMs noted postoperative improvements compared to baseline, although many studies included concomitant procedures. Implant failure rate was reported in five studies (29.4%), and the reoperation rate was reported in one study (5.9%) [7, 9, 11, 13, 21].

### Radiographic outcomes following scaffold implantation

Several studies examined the radiographic impact of using these scaffold implants. Li and colleagues examined polyurethane‐based scaffolds and concluded that while PROMs improved, there was a morphological deterioration of the implant seen on MRI at the most recent follow‐up in the studies included [15]. They were unable to conclude whether that deterioration was clinically relevant. Ranmuthu and colleagues also radiographically examined the use of CMI and Actifit and found possible chondroprotective effects, as assessed by MRI [20]. Similar to Li and colleagues, however, they were unable to conclude any clinical translation due to differences in the included study methodology and small sample sizes. Like Li and colleagues, Shin and colleagues concluded that meniscal scaffolds seem to be a viable option for patients with partial meniscal defects but found worsening articular cartilage and absolute meniscal extrusion, as assessed by MRI [25]. Despite these findings, they could not definitively establish the clinical relevance of these changes. However, Zaffagnini and colleagues and Warth and colleagues found that compared to initial MRI evaluations, later evaluations demonstrated that the scaffolds were more similar in appearance to normal menisci [32, 34]. Likewise, Filardo and colleagues found that both CMI and Actifit promoted tissue healing based on MRI findings [7]. However, they also noted the poor quality of evidence included in their review.

## DISCUSSION

The aim of this second part of our two‐part umbrella review was to identify literature gaps and suggest future research directions. Similar to the first part, our analysis reveals a significant increase in interest in scaffold‐based approaches, as evidenced by the growing number of reviews published in recent years [33]. However, many of these reviews lack of standardized reporting and rigorous methodologies, which has limited their overall quality. Future reviews would benefit from a more comprehensive approach, including a thorough assessment of bias risk, adherence to PRISMA guidelines and careful consideration of bias risk during evidence synthesis.

Interestingly, the journals in which these reviews were published differ from those focused on MAT [33]. For example, there were no reviews about scaffolds published in AJSM, whereas AJSM was the most common journal for MAT reviews. This discrepancy could be attributed to factors such as the more experimental nature of scaffold implants and their limited use in the United States. As long‐term data and FDA approvals for these implants become more widespread, we may see an increase in publications similar to those for MAT. While several reviews have addressed surgical techniques for MAT, there continues to be a lack of reviews on optimal surgical techniques for scaffold implants [5, 12, 17].

The reviews included have shown promising short‐term PROMs; however, they have also concluded that there is insufficient evidence to fully support the use of these implants. One area of future research lies in understanding the clinical implications of radiographic changes seen in long term follow‐up of scaffolds implant. Although multiple reviews have noted morphological changes during postoperative follow‐ups, none of them have been able to establish any clinical relevance [15, 25, 34]. Future studies and reviews can focus on understanding both the long‐term outcomes of these implants and the implications of these changes. Although there have been several studies focused on risk factors for transplant failure after MAT, there has been no comparable study focused on risk factors for implant failure in scaffolds. A better understanding of these risk factors can help guide patient selection.

There is an ongoing need to establish clear guidelines for the use of scaffolds in treating meniscal deficiencies. While some reviews suggest avoiding their use in osteoarthritis cases altogether, other studies indicate potential benefits based on patient characteristics like age and activity level [6, 7]. Scaffold‐based repairs cannot be used for complete meniscectomy as they require the presence of both anterior and posterior horns [7, 21]. Further research and reviews are needed to establish criteria for patient selection in scaffold‐based approaches. Future meta‐analyses and randomized controlled trials comparing outcomes of patients with osteoarthritis treated with scaffold‐based approaches to those without may help better inform the management of these patients.

It is important to highlight that these scaffolds have limited availability in certain regions, particularly with regards to FDA approval in the United States. However, despite this limitation, synthesizing the literature on meniscal scaffold‐based approaches remains crucial for several reasons. First, it provides valuable insights into the current state of research and clinical practice in this area, helping to identify gaps in knowledge and areas for future research. Second, it can guide clinical decision‐making by summarizing the available evidence on the effectiveness and safety of these approaches. Finally, it can inform regulatory bodies and healthcare policymakers about the need for further research and the potential benefits of making these scaffolds more widely available. Therefore, despite their limited availability, synthesizing the literature on meniscal scaffold‐based approaches is essential for advancing our understanding and improving patient care in this field.

While we acknowledge the limitations outlined in Part 1 of our review [33], it is crucial to emphasize several key limitations. The heterogeneity among the included reviews, particularly regarding patient populations, surgical techniques and outcome measures, introduces variability in the conclusions and may limit the generalizability of the findings across different patient groups and surgical approaches. The complexity of interpreting findings from multiple reviews with diverse methodologies and outcomes can lead to potential misinterpretation. Furthermore, the variability in the quality of the included reviews, especially in terms of standardized reporting and rigorous methodologies, could impact the overall reliability of the umbrella review's conclusions. These limitations underscore the importance of cautious interpretation when considering the results of this umbrella review.

## CONCLUSION

Meniscal scaffold‐based approaches offer an option for managing meniscal deficiency, particularly in cases where preservation techniques are not feasible. However, the current evidence base is limited by methodological shortcomings and a lack of clear guidelines for patient selection and surgical technique. While short‐term outcomes following scaffold implantation are generally positive, with improvements in pain relief and function reported in many studies, there are concerns regarding relatively high failure rates. Future research should focus on conducting well‐designed randomized controlled trials with long‐term follow‐up to further elucidate the benefits and indications of these techniques in clinical practice. Efforts should also be made to develop consensus guidelines to standardize the use of meniscal scaffolds and improve patient outcomes. Despite their limited availability, synthesizing the literature on meniscal scaffold‐based approaches is crucial for advancing our understanding and improving patient care in this field.

## AUTHOR CONTRIBUTIONS

All authors contributed to the idea, initiation, execution and revision of the study. Kevin A. Wu wrote the original draft. Stephanie Hendren performed the literature search. Lulla V. Kiwinda, Aaron D. Therien and Christian J. Castillo collated data. Kevin A. Wu, Lulla V. Kiwinda, Aaron D. Therien analyzed the data. Jason S. Long, Annunziato Amendola, Brian C. Lau conceptualized the project and gave critical feedback. All authors agreed on the order of authorship prior to manuscript submission. The authors read and approved the final manuscript.

## CONFLICT OF INTEREST STATEMENT

The authors declare no conflict of interest.

## ETHICS STATEMENT

This is a review study. The Duke Health Research Ethics Committee has confirmed that no ethical approval is required.

## Data Availability

Data sharing is not applicable to this article as no new data were created or analyzed in this study.
